# Obesity prevalence and associated risk factors in outdoor living domestic horses and ponies

**DOI:** 10.7717/peerj.299

**Published:** 2014-03-20

**Authors:** Sarah L. Giles, Sean A. Rands, Christine J. Nicol, Patricia A. Harris

**Affiliations:** 1 University of Bristol, School of Veterinary Science, Langford, Bristol, UK; 2 University of Bristol, School of Biological Sciences, Bristol, UK; 3 Equine Studies Group, WALTHAM Centre for Pet Nutrition, Melton Mowbray, Leicestershire, UK

**Keywords:** Horse, Pony, Obesity, Body condition, Season, Prevalence, Risk factors, Equine

## Abstract

**Reasons for performing study.** The prevalence of obesity in companion animals, including horses and ponies has risen drastically in recent years and risk factors have been little investigated. Horses are unique amongst companion animals in that many are outdoor-living and forage independently on pasture; they also have a dual utility and companionship role. The body condition of wild and free-living equines is known to vary seasonally, yet previous estimates of the prevalence of obesity and associated risk factors in domestic animals do not consider this. Most previous studies were conducted during the summer months when pasture quality is greater and obesity prevalence is likely to be highest. In addition, many previous estimates do not use validated body condition scoring methods and rely on owner reporting.

**Objectives.** To examine the prevalence and risk factors predictive of equine obesity at both the end of winter and the end of summer, in a domestic population of leisure horses with daily access to pasture. Using validated body condition scoring methods and a single, trained observer.

**Methods.** Body condition and belly girth measurements were taken at the end of winter and during the summer in a population of leisure horses (*n* = 96) with outdoor pasture access for ≥6 h per day. Risk factor information was obtained by two owner questionnaires and analysed statistically using a mixed effects logistic regression model. The dependent variable was obese (BCS ≥ 7/9) or non-obese (BCS < 7/9). Risk factors associated with seasonal change in belly girth were also explored using a mixed effects linear regression model.

**Results.** Obesity prevalence rose significantly from 27.08% at the end of winter to 35.41% during summer (*p* < 0.001). Breed was the risk factor most strongly associated with obesity (*p* < 0.001). Supplementary feed was not a strong predictor and there was no association with low intensity structured exercise. As winter BCS increased, the percentage seasonal change in belly girth decreased.

**Conclusions.** Obesity prevalence differed between winter and summer in domestic equines. Supplementary feed and low intensity structured exercise in equines living outdoors for ≥6 h per day had limited or no effect on obesity levels. Seasonal variation in body condition was lower in obese equines.

**Potential relevance.** It is important to consider season when studying equine obesity and obesity-associated disorders. Risk factor analysis suggests preventative measures may need to be breed specific. The metabolic implications of a lessened seasonal change in body condition in obese animals, warrants investigation.

## Introduction

Obesity is an increasingly common non-communicable disease problem in equines in developed countries (Thatcher et al., 2008; Wyse et al., 2008). Equine obesity has serious health and welfare implications, being associated with an increased risk of insulin resistance (Hoffman et al., 2003), laminitis (Geor, 2008; Treiber et al., 2006) (a severe and debilitating condition of the hoof, which can cause chronic lameness) and osteoarthritis (Schlueter & Orth, 2004). Some now consider it the most important welfare issue affecting equines in western countries (Owers & Chubbock, 2013).

Table 1 summarises previous equine obesity prevalence estimates. Some rely upon owner reporting, which is known to be inaccurate (Wyse et al., 2008; NAHMS, 1998) and the body condition scoring methods used and definitions of overweight and obese vary. The Henneke (Henneke et al., 1983) nine point body condition score (BCS) is relatively accurate as an assessment of body fat at BCS < 7, but due to an exponential relationship with actual body fat content, categories above 7 cannot be differentiated and are better classified as a single ‘obese’ category (Dugdale et al., 2011). Using validated categories assessed by a single trained observer, this presents an opportunity to obtain an updated and more accurate prevalence estimate.

**Table 1:** Summary of previous equine obesity prevalence estimates.

Study	Region	Sample size	Condition scoring method(s) used	Observer	Time of year	Definition of obese	% under weight	% moderate weight	% over weight	% obese
NAHMS (1998)	USA	2904	-	Owner reported	-	-	-	-	-	1.4%
Thatcher et al. (2008)	Virginia (USA)	300	Henneke et al. (1983) 9 point BCS, BMI, neck circumference to height ratio	Multiple trained	Summer	BCS of 7.5–9/9	2%	47%	32%	19%
Wyse et al. (2008)	Scotland	319	Webb & Weaver (1979) 6 point BCS	Multiple trained and owner reported	Summer (June/July)	BCS of 5 or 6/6	2%	53%	-	45%
Stephenson, Green & Freeman (2011)	Leicestershire and Nottinghamshire (England)	160	Caroll & Huntington (1988) 5 point BCS	Owner reported	Summer (August/ September)	BCS > 3/5^*^	1.9%	77.5%	20.6%	-
Harker, Harris & Barfoot (2011)	England	331	Henneke et al. (1983) 9 point BCS	Multiple trained	Summer (July)	BCS of 7–9/9	3%	30%	41%	21%
Ireland et al. (2012)	United Kingdom (Geriatric horses)	200	Caroll & Huntington (1988) 5 point BCS	Multiple trained	Summer (June–October)	BCS > 3/5^*^	4.5%	69.5%	26%	-

**Notes.**

* In these studies overweight and obese were combined into a single category.

Additionally, where time of year is known, previous studies have been conducted solely during the summer months (Table 1). This complicates interpretation, as body condition (especially in free-living horses) varies seasonally (Dugdale et al., 2011; Scheibe & Streich, 2003). Plausibly the prevalence of obesity may vary year-round—something that has not been previously investigated.

Seasonal body condition change in equines is an adaptive mechanism which aided survival in a sparse, highly seasonal, ancestral environment (McGregor-Argo, 2009). As a result the physiological satiety mechanisms of horses and ponies respond to photoperiodic changes in day length (McGregor-Argo, 2009). Increased appetite and metabolic rate (Arnold et al., 2006) during the summer result in fat deposition when high quality forage is available. In winter a decreased appetite and reduced metabolic rate (‘hypometabolism’) increases efficiency and conserves energy when food is scarce (Dugdale et al., 2011; Brinkmann, Gerken & Riek, 2012; Kay & Staines, 1981). These cycles result in seasonal fluctuations in body condition.

The principal, proximal cause of obesity is an energy imbalance, where an oversupply of energy from the diet exceeds energetic expenditure. But the distal risk factors influencing both parts of this imbalance have been little investigated in equines.

The aim was to investigate the prevalence and risk factors for obesity in partly and totally outdoor-living leisure horses and ponies in part of the UK, owned by local riding club members or local charitable organisations (see methods). The objectives were: (1) to use a single trained observer to assess obesity prevalence in the study population during both winter and summer; (2) to examine risk factors associated with obesity in the study population; and (3) to examine seasonal body condition changes within individual animals using a measure of belly girth.

## Materials and Methods

### Animals

Horses were selected from a sampling frame of leisure horses and ponies kept by local charitable organisations or kept by riding club members who attended a membership renewals meeting in January 2011 and consented to participate.

Inclusion was restricted to horses and ponies living in herds of two or more individuals which grazed together for at least six hours a day and had been living together for at least one month. In addition, individuals had to be present in the same herd for both winter and summer measurements.

### Study design

The study was carried out in North Somerset, England. The initial set of measurements were taken on 127 horses and questionnaires administered between 5th February and 24th March 2011. Our study population concerns the 96 (*n*) study individuals from this initial set of 127 who were then also available for a second set of summer measurements and questionnaires, administered between 20th July and 1st September 2011.

Grass began to grow at the end of March at which time recruitment was stopped. There were 26 total herd groups. A cluster sampling strategy was used; horse herds were randomly selected from the list of owners willing to participate and all horses measured from that herd.

The work was approved by the University of Bristol Ethical Review Group (University Investigation Number UB/10/049).

### Outcome measures

#### Body condition score

Initial body condition measurements were taken and scored using the Henneke et al. (1983) nine point body condition score as adapted by Kohnke (1992), ranging from 1 (emaciated) to 9 (obese). Six anatomical regions of the horse were scored on the nine-point scale: the mean value was calculated and rounded up to obtain a single whole-number score (Kohnke, 1992). Half measures were not used.

Based on the findings of Dugdale et al. (2011), obesity was defined as a binary variable for risk factor analysis, with individuals scoring a BCS of 7, 8 or 9 classified as ‘obese’; and those scoring 6 or less classified as ‘non-obese’. Where the outcome variable was ‘case of obesity during the study’ versus ‘no case of obesity during the study’, i.e., the case of obesity could have been at the end of winter, at the end of summer or both.

#### Belly girth

Belly girth was defined as the widest point on the animal, around two thirds of the way between the point of the shoulder and the point of the hip (Dugdale et al., 2010). A measure of belly girth (centimetres) was taken from each animal on both sampling occasions (*n* = 96, see Fig. 2). Percentage change in belly girth between winter and summer measurements was calculated for each individual animal, and this was the outcome variable used in risk factor analysis.

For the analysis of the relationship between BCS and seasonal change in belly girth (Fig. 2) Henneke categories 1 to 6 were differentiated, while 7 to 9 were condensed into a single ‘obese’ category.

Both BCS and belly girth measures were scored and recorded by a single trained researcher (SLG).

### Explanatory variables

Height was recorded at the withers using a measuring stick with spirit level. Information on all other explanatory variables was obtained from owners, via questionnaires (see Supplemental Information), that were piloted on a small sample of horse owners prior to the start of the investigation. The winter questionnaire focused on baseline data and management, including age, breed, sex, height, number of years owned, hours at pasture, additional feed, type and duration of structured exercise, rug routine, grazing setup and restrictions, worming frequency and pasture maintenance. The summer questionnaire was similar but additionally detailed any changes in management due to season, such as whether grazing was further restricted and changes in the herd makeup since the winter visit. Both questionnaires included a detailed breakdown of food and exercise parameters. Different food types were classified and the quantity of each component fed per day was estimated by owners and then grouped for analysis. The total exercise hours per week and the estimated time spent in different activities per hour of ridden exercise was recorded. Inclusion was not restricted by age, so that the effect of age could be fully explored within the analysis, however a measure of belly girth was not taken in very young animals (<2 years, *n* = 3) as any seasonal changes would be mostly due to growth. Questionnaires took around ten minutes to complete and consisted mainly of closed questions. All owners completed the questionnaire alone in their own time, to remove observer influence.

### Strategy of statistical analysis

All data analysis was carried out using *Stata* 11.2 (Statacorp, Texas).

#### Data handling

Initially the data were checked and any missing values noted. Missing values were dealt with using complete case analysis, where all units with incomplete data were removed from the model. Transformations were considered where appropriate but not required for final model variables.

Height was recoded into a binary variable to compare potential differences between horses (above 148 cm, equivalent to 14.2 hh) and ponies (148 cm and below) but additionally analysed as an ordered categorical or continuous variable. Breed was coded into seven categories for analysis based on common breed groupings (Goodall, 1973): (1) native ponies (except Shetland), this included Welsh ponies, Exmoor, Dartmoor and New Forest ponies, (2) Shetland ponies and miniature breeds, (3) lightweight breeds, including Arabian and Thoroughbred types, (4) heavyweight draught horses, including shire horses and other heavy breeds, (5) cob types, including both Welsh and mixed breed cobs, (6) sports horse breeds, including Irish Draught and warmblood breeds and (7) ‘other’, where the horse or pony did not fit into an obvious breed category.

Age was analysed as a continuous variable, but also split into youngster (≤4 years) and non-youngster (>4 years), this is the usual age at which horses reach adult size.

Quantity of both supplementary feed and hay or other forage was coded into categories for analysis, and also analysed as a binary variable (any *versus* none). The categories of supplementary feed represented: category 1, no supplementary feed at all; category 2, no energy providing feed (concentrate) but vitamin and mineral supplements given; category 3, <1.5 kg day^−1^ of ‘concentrate’ energy providing feed; category 4, ≥1.5 kg day^−1^ of concentrate, energy providing, feed. These categories were analysed separately for horses and ponies, to account for the size of the animal. Hay was split into 3 categories, none, small amount (<5 kg), and large amount (≥5 kg).

#### Data analysis

Initially risk factor variables were analysed individually, using either *χ*^2^ tests, analysis of variance (ANOVA) or regression techniques (all two-tailed). Associated variables were then put forward into a multivariable mixed effects logistic regression model for the outcome obese/non-obese, and a multivariable linear mixed effects model for the outcome percentage change in belly girth. For both models a screening *p*-value of ≤0.07 was used. Co-linearity was considered through cross tabulation and pairwise correlations, and where necessary, the variable which added the most to the model by assessment using the likelihood ratio test, was retained.

The regression models were forward-fitted using a positive stepwise approach, based on strength of univariable association, whereby those variables with the strongest univariable association are added first, starting from a minimal model. Variables were retained on the basis of *p*-values (indicating overall significance when controlling for other model variables) and also contribution to the model fit, assessed using the likelihood ratio test. The clustered study design was controlled for by including herd group in the model as a random effect. Model residuals and other assumptions were checked.

The category BCS = 3 was removed when exploring the association between the two outcome variables, BCS and seasonal change in belly girth, as there was only one individual in it. This individual was removed from the analysis here.

## Results

### Descriptive statistics

All mean values are reported alongside the standard error of the mean, unless otherwise stated.

The response rate to both questionnaires was 100%, 96 individuals were measured in both seasons, within 26 herds. Herd size ranged from 2 to 17 individuals, with a mean herd size of 7.4 ± 0.7.

77% of the study population lived outdoors for 24 h a day in winter and 81% in summer. Those that were stabled for part of the day spent on average, 11.82 ± 0.55 h at pasture during winter and 13.66 ±0.36 h during summer.

The median height was 142 cm (14.1 hh, 25th percentile 125 cm, 75th percentile 155 cm). The median height of ponies was 130 cm (12.3 hh, 25th percentile 119 cm, 7th percentile 137 cm) and of horses was 157 cm (15.2 hh, 25th percentile 152 cm, 75th percentile 165 cm). The mean age in winter was 10.60 ± 0.60 years, ranging from 8 months to 28 years. There were 24 youngsters and 69 adult horses (3 where age was unknown). The mean age of youngsters was 2.8 ± 0.16 years. In total there were 46 horses and 50 ponies.

94% of the horses and ponies considered in the study were fed some form of supplementary feed during winter, 92% were fed additional hay and 36% were fed energy supplying ‘concentrates’. During summer, the proportion of horses and ponies fed supplementary feed fell to 62%, and only 29% were fed additional hay. During winter 1% of ponies and 13% of horses received ≥1.5 kg of concentrate feed per day. During summer, 1% of ponies and 4% of horses received ≥1.5 kg of concentrate feed per day. During winter, 9% of horses and ponies were fed just vitamins and minerals (without other supplementary feed) and 11% were fed just these in summer. 64% undertook no form of structured exercise during winter months; this was similar (68%) during the summer. 72% of ponies and 61% of horses undertook no structured exercise. Horses and ponies that experienced structured exercise carried out on average 4.84 ± 0.29 h per week during winter and 6.14 ± 0.30 h per week during summer.

Comparing ridden horses and ponies (*n* = 49 winter, *n* = 36 summer), horses carried out 7.47 ± 1.81 h of exercise per week during the summer, compared with 4.31 ± 1.08 h per week in ponies (*F*_1,34_ = 13.21, *p* < 0.001). During the winter, horses carried out 5.45 ± 0.58 h per week exercise, and ponies, 3.5 ± 0.62 h per week (*F*_1,47_ = 7.72, *p* = 0.008).

Notably, the variability in BCS between individuals within herds was 1.79 BCS (range 0–4 BCS).

### Prevalence of obesity

At the end of winter the prevalence of obesity was 27.08% (95% CI [18.28–35.80]) and at the end of summer the prevalence of obesity was 35.41% (95% CI [25.91–44.91]). The number of individuals that experienced at least one case of obesity during the study was 41.76% (95% CI [31.96–51.56]). Of those without obesity at the end of the winter months (70/96), 14 individuals became obese by the end of the summer, giving an incidence of 20% over a 6 month period. Only 6 individuals (6.25%) were obese at the end of winter, but not obese at the end of summer.

**Figure 1:**
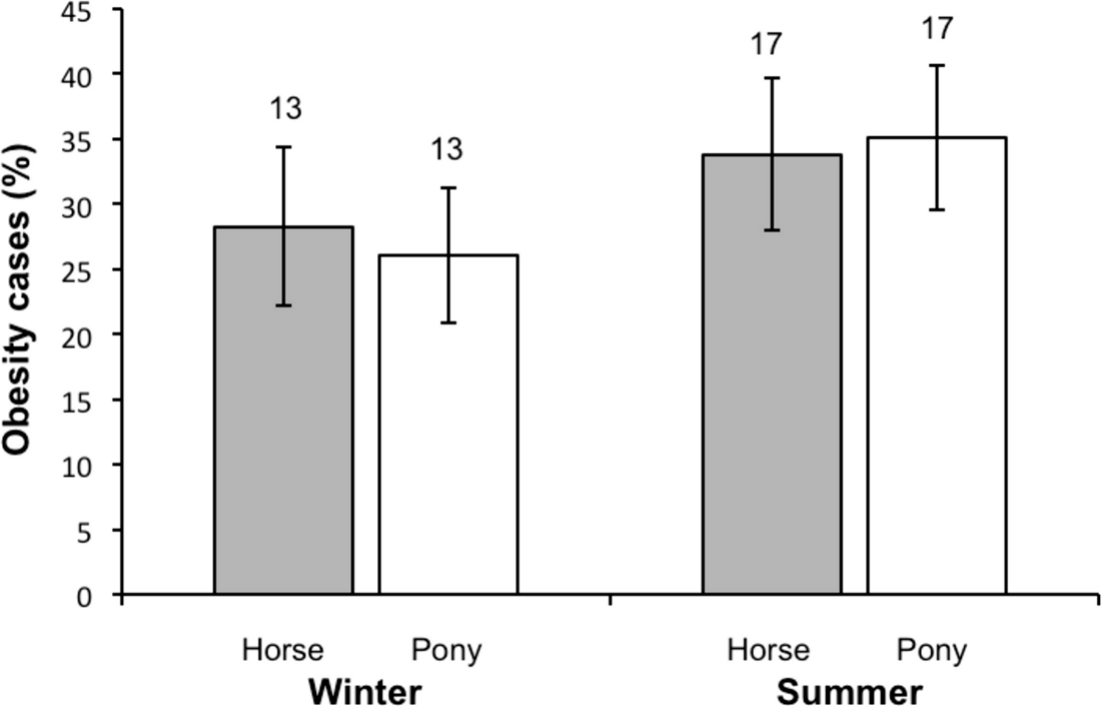
A comparison of obesity prevalence in horses and ponies between winter and summer. Winter: RR (Risk Ratio, Horses/Ponies) = 1.06, 95% CI [0.67–1.67] *p* = 0.80. Summer: RR (Horses/Ponies) = 1.07, 95% CI [0.70–1.64], *p* = 0.76.

There was no significant difference in obesity prevalence between horses and ponies either at the end of winter or the end of summer (Fig. 1).

### Risk factors for obesity

Univariable associations with obesity included breed (*F*_6,89_ = 4.61, *p* < 0.001), gender (${\chi }_{2}^{2}$ = 6.58, *p* = 0.04), whether or not the horse or pony was fed additional food during winter (${\chi }_{1}^{2}$ = 6.89, *p* = 0.009), had a new injury between winter and summer measurement (${\chi }_{1}^{2}$ = 5.30, *p* = 0.02), the individual was fed treats or titbits during winter (${\chi }_{1}^{2}$ = 5.76, *p* = 0.02), a new companion between winter and summer (${\chi }_{1}^{2}$ = 3.29, *p* = 0.07), the individual is a youngster (≤4 years, ${\chi }_{1}^{2}$ = 4.85, *p* = 0.03) and whether the individual was fed sugarbeet during winter (${\chi }_{1}^{2}$ = 4.34, *p* = 0.04).

The final multivariable logistic regression model is presented in Table 2, and controls for clustering by herd group. This final model contains the risk factors breed, whether the individual is a youngster and new injury between winter and summer. Compared to lightweight breeds as baseline, native cobs have 13.61 times the odds of obesity (*p* = 0.006) and native ponies also appear to be at increased risk with 2.3 times the odds, though this was not significant. Young horses were 0.82 times less likely to be obese than older horses (*p* = 0.01), and those which had a new injury between winter and summer measurements had 5.53 times the odds of obesity compared to those without a new injury (*p* = 0.01).

**Table 2:** Multivariable mixed effects logistic regression model showing risk factors associated with obesity (BCS ≥ 7).

**Risk factor**	**Total number** **of equines (%)**	**Number of** **obesity cases (%)**	**Odds ratio**	**SE**	**95% CI**	*p*
**Breed**						
Native ponies (except Shetland)	30 (31.2)	12 (30.0)	2.30	1.60	0.58–9.01	0.23
Lightweight (Baseline)	25 (26.0)	5 (12.5)	1	-	-	-
Heavyweight/Draught^*^	4 (4.1)	4 (10.0)	-	-	-	-
Cob types	12 (12.5)	8 (20.0)	13.61	13.03	2.08–88.93	0.006
Sports horse	13 (13.5)	3 (7.5)	0.56	0.52	0.09–3.47	0.54
Shetland and Miniature	4 (4.1)	1 (4.0)	1.14	1.72	0.06–21.75	0.93
Other	8 (8.3)	7 (17.5)	27.60	35.26	2.25–337.52	0.009
**Youngster**						
>4 years, not youngster (baseline)	69 (71.8)	32(80.0)	1	-	-	-
≤4 years, youngster	24 (25.0)	5 (12.5)	0.18	0.13	0.46–0.73	0.01
**New injury**						
No new injury (baseline)	76 (79.1)	28 (70.0)	1	-	-	-
New injury	18 (18.8)	12 (30.0)	5.53	3.80	1.44–21.30	0.01
	96 (100)	40 (41.76)				

**Notes.**

* Low sample size in this category.

### Seasonal changes in body condition

**Figure 2:**
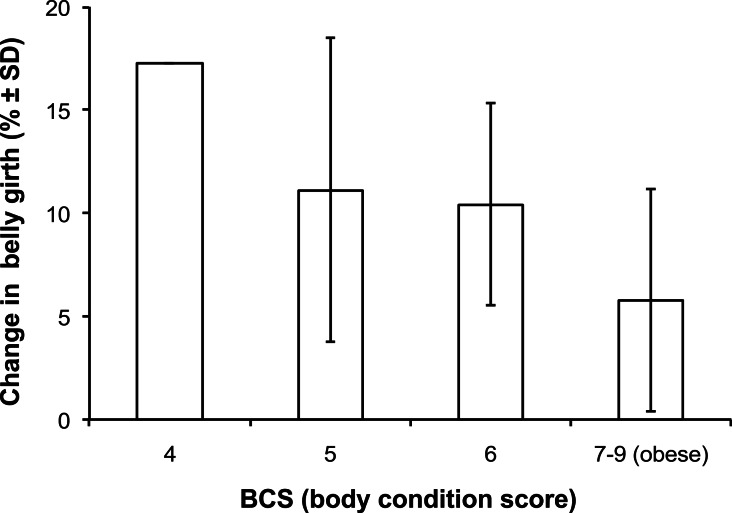
Summary of the association between winter body condition score and mean percentage seasonal change in belly girth.

Seasonal changes in body condition were assessed using the outcome ‘percentage change in belly girth’ (*n* = 93). The mean between season percentage change in belly girth was an increase of 10.12 ± 0.67%, with a range between −6.67% (decrease) and 28.65%. A one-way ANOVA revealed a strong relationship between winter BCS and mean percentage seasonal change in belly girth (Fig. 2, *F*_3,88_ = 10.62, *p* < 0.001) i.e., as winter body condition score increased, the percentage seasonal change decreased. The repeatability of the belly girth measure between seasons, assessed using a Bland-Altman plot, showed a 18.55 cm mean difference between winter and summer belly girth measures, showing a strong tendency for summer belly girth measurements to be higher than winter measures by an average of 18.55 cm (95% CI [20.88–16.22]).

Risk factors associated with seasonal percentage change in belly girth included breed (*F*_6,86_ = 5.51, *p* < 0.001), age (*t*_89_ = −2.55, *p* = 0.01), height (*t*_92_ = −5.24, *p* < 0.001), grass restriction method (*F*_5,76_ = 3.58, *p* = 0.006), seasonal change in feed regimen (*F*_3,86_ = 3.10, *p* = 0.03) and whether or not owners control grass intake (*F*_1,89_ = 6.47, *p* = 0.01).

Table 3 shows the multivariable mixed effects linear regression model for risk factors associated with percentage seasonal change in belly girth. Lightweight horses appear to have the largest seasonal change in belly girth. All other breeds show a lower percentage seasonal change when compared to lightweight breeds as baseline. Horses and ponies whose owners implemented a complete change in feeding regimen had a 6.71% higher seasonal change in belly girth, compared to horses and ponies who did not experience a change in feed regimen. This is probably due to the owners of more obese and obesity prone individuals attempting to control this during summer months, and therefore an anomaly due to cause and effect. As these were outdoor living animals, it is no surprise that controlling grass intake had the largest effect in reducing the seasonal percentage change in belly girth. Compared to those individuals whose grass intake was not controlled, the seasonal belly girth change was 5.42% lower in those whose grass intake was controlled by owners. Grass intake was restricted using a number of methods (moving horses between paddocks, grazing muzzle, restricted grazing time or removal from pasture) and ‘grass restriction method’ also showed a multivariable association with percentage change in belly girth, but this variable was removed from the final model due to collinearity with the ‘controlling grass intake’ variable, i.e., they explained the same variation in belly girth.

**Table 3:** Multivariable mixed effects linear regression model showing risk factors associated with percentage change in belly girth between winter and summer measures.

**Risk factor**	**Total number** **of equines** (%, ***n*** = 93)	**Coefficient**	**SE**	**95% CI**	*p*
**Breed**					
Native ponies (except Shetland)	28 (30.1)	−3.67	2.13	−7.84–0.51	0.08
Lightweight (baseline)	25 (26.9)	0	-	-	-
Heavyweight/Draught	4 (4.3)	−3.99	3.30	−10.46–2.48	0.23
Cob types	11 (11.8)	−3.80	1.91	−7.56–−0.06	0.05
Sports horse	13 (14.0)	−0.57	1.78	−4.07–2.92	0.75
Shetland and Miniature	4 (4.3)	−11.1	4.48	−19.96–−2.41	0.01
Other	8 (8.6)	−3.90	2.14	−8.09–0.30	0.06
Height (cm)	93 (100)	−0.23	0.06	−0.34–−0.12	<0.001
**Seasonal change in feeding regimen**					
No change	12 (13.3)	0	-	-	-
Reduction in quantity only	13 (14.4)	3.47	2.15	−0.74–7.68	0.11
Change parts	56 (62.2)	2.92	1.75	−0.52–6.37	0.09
Complete change	9 (10.0)	6.71	2.38	2.04–11.38	0.005
**Control of grass intake** ^*^					
No	12 (13.2)	0	-	-	-
Yes	79 (86.8)	−5.42	1.76	−8.88–−1.97	0.002
**Constant**		47.11	8.77	29.92–64.29	<0.001

**Notes.**

* Grass restriction method also showed evidence of association but this variable was removed due to colinearity.

Additionally there was strong evidence for a difference in BCS within the same individuals between seasons (*n* = 96, Fisher’s exact *p* = 0.02). Repeatability of the Henneke BCS using the same observer and same individuals but in different seasons was poor (agreement = 44.79%, *kappa* = 0.27).

## Discussion

Seasonal differences in the prevalence of equine obesity are reported here for the first time by assessing the body condition study horses and ponies at two key seasonal time points. This gives some indication of the potential seasonal variability in equine obesity, within the same population of animals, throughout the year. The study results refer to animals that were at pasture for a minimum of 6 h per day, in which seasonal trends were likely to be clearest. An obesity prevalence of 27% at the end of the winter months had risen significantly to 35% by the end of the summer. This highlights the importance of accounting for season when studying obesity and obesity related disorders, especially in outdoor-living equines. Prevalence estimates are within the range suggested by previous studies (Wyse et al., 2008; Harker, Harris & Barfoot, 2011; Stephenson, Green & Freeman, 2011), but previous studies do not account for this seasonal variation.

At the end of winter, outdoor-living horses and ponies are likely to be in their lowest state of body condition, and during mid-summer they are likely to be in their highest. Seasonal variation in body condition is in line with the seasonality of food supply and also represents a historical winter survival strategy. Wild or feral living horses and ponies store fat during the summer months to aid survival during harsh winters, which results in this fluctuating body condition through the year (Scheibe & Streich, 2003; Arnold et al., 2006; Fuller, Cox & Argo, 2001).

Certain breeds appear to be more at risk of obesity and associated disorders (Johnson et al., 2010). Breed was strongly associated with equine obesity, with native UK breeds most likely to be obese. Native UK breeds are particularly ‘thrifty’ (Treiber et al., 2006) and can survive on sparse, seasonal, fibrous grasses, this being the forage that their digestive tract has evolved to utilise (Janis, 1976). In contrast to the ancestral environment, for many native leisure horses food is plentiful year-round, even in outdoor-living animals, due to a combination of high quality ‘improved’ pasture, designed to support high production farm animals, and the supplementary feeding of forage in winter. As a result these more ‘thrifty’ breeds may be more at risk of obesity.

There were also differences in management related to breed. The percentage of ponies experiencing no structured exercise was higher than for horses suggesting that pony breeds are more commonly kept for companion purposes. However obesity levels were very similar in both horses and ponies, no significant difference was found between them in either season, thus challenging a common myth that ponies are more at risk of becoming obese (McGregor-Argo, 2009), at least in animals spending a large amount of time at pasture. It appears instead that breed is the important factor, not height, with native UK breeds, both cobs and ponies, both at greater risk.

Fewer young horses were obese than older horses. This is probably the result of the increased energy requirements of growth and a greater energy expenditure when out at pasture, as young horses may be more active than older horses. Young horses experience increased growth rates during the summer months due to better nutrient availability (Pagan, Jackson & Caddel, 1996), meaning they may be at less risk of developing obesity during this time. It is also possible that older horses may have had an energy-exercise imbalance for a much longer time period, possibly further contributing to the apparent difference in obesity susceptibility between young and adult animals.

New injuries incurred between the winter and summer measurements were the final risk factor in our multivariable model. A discussion regarding this association justifies consideration of cause and effect. It may be that obese individuals are more likely to become injured, due to a greater mechanical load upon joints (6), or associated metabolic complications (3, 4, 5). Alternatively individuals that are injured and cannot be ridden may carry out less exercise and thus be more prone to obesity. However, exercise did not show any evidence of association with obesity risk.

A measure of belly girth was taken as a more sensitive indicator of short-term seasonal changes in body fat (Dugdale et al., 2011). Winter body condition score was strongly associated with percentage seasonal change in belly girth (*p* < 0.001), as animals become obese, the variation in body condition between winter and summer lessens. This reduction in seasonal change may reflect a decrease in appetite as horses become obese. Strong seasonal trends in both appetite and associated body condition have been observed in feral horse populations (Scheibe & Streich, 2003) and are thought to be photoperiodically entrained (Scheibe & Streich, 2003; Fuller, Cox & Argo, 2001). Dugdale et al. (2011) showed seasonal body condition and appetite changes in a small group of native breed mares. In this current study the largest seasonal changes were seen in individuals whose access to pasture was not controlled or restricted, all of these individuals increased in belly girth during the summer months, indicating that natural seasonal changes in body condition are still present in domestic, leisure, populations. Belly girth was probably marginally higher in the summer due to greater grass availability and a subsequent increase in gut fill; however the authors felt it was still a useful incremental measure of short term changes in overall body condition, which may be otherwise missed by the Henneke 9 point score.

The management of domestic horses places an emphasis on keeping horses in a constant ‘good’ (BCS = 5) condition year-round, with little tolerance for natural patterns of seasonal variation. Based on natural physiological survival strategies, horses with an overweight or obese body condition at the end of summer can withstand poorer pasture quality and winter weight loss, which may not be widely appreciated within the domestic environment. Conceivably, owner perception of body condition has been skewed upwards due to the high prevalence of overweight and obese horses/ ponies within the UK population (Wyse et al., 2008). The welfare implications of year round adiposity *versus* seasonal adiposity are currently poorly understood. The relative metabolic consequences of summer obesity, followed by winter weight loss, versus year-round moderate adiposity, are unknown, but this would warrant future investigation.

Although the study population consisted of predominantly outdoor-living equines, it was expected that supplementary feeding, particularly of energy providing concentrates, would be a strong risk factor, due to the suggestion that over-feeding companion animals is largely responsible for their obesity (D’Eath et al., 2009; McGreevy et al., 2005). Relying on owners’ estimates of supplementary feed is not ideal, but some of this influence was removed by condensing amounts fed into broad and carefully considered categories for analysis. There could be value exploring supplementary feeding where energy content and exact amounts could be accurately measured, but this was beyond possibility with this particular study population due to the *post hoc* nature of the data reporting. There was some evidence that supplementary feeding during winter was associated with obesity in the univariable analysis, which may warrant future investigation. However the lack of association with other feed-related parameters was notable and suggests that when horses are living outdoors, additional feed plays only a small role in their likelihood of becoming obese. It is likely that grass consumption is the most important factor influencing obesity risk in outdoor living animals and this should be considered in more detail in future work.

If we consider the amount of grass consumed through normal grazing behaviour (Longland & Byrd, 2006), the provision of supplementary forage and energy providing feed may in fact have a small comparative influence in circumstances where grass intake is not restricted. Whether or not owners controlled grass intake did show an association with seasonal body condition change, with those individuals whose access to grass was unrestricted showing the largest belly girth increase from winter to summer. Pasture presents both spontaneous exercise opportunities and an uncontrolled source of food. As individuals lived outdoors for at least 6 h per day (mostly longer), they would be able to consume a significant amount of grass during such a period. Previous research indicates that ponies can eat up to 4.9% of body weight in dry matter (Longland, Ince & Harris, 2011) over a 24 h period and up to 1% of body weight (Ireland et al., 2012) when out at pasture for only 3 h, indicating that in some individuals, considerable amounts of grass can be ingested in even a short time period out at pasture. Another study estimated that by the end of 6 weeks ponies were ingesting 40% of their dry matter intake during 3 h pasture turnout (Goodall, 1973). All horses and ponies were at pasture for relatively long periods in this study due to the inclusion criteria, this may explain why there was no direct association between time spent at pasture and risk of obesity in this study.

Feeding behaviour of equines at pasture may also vary with breed. Native UK breeds will be more adapted to survive in the UK winter climate and thus may be more successful foragers in harsh winter climates, and more able to conserve energy during winter months. More lightweight breed types such as Arabians and Thoroughbreds may spend less time foraging and not have the same ‘thrifty’ winter survival mechanisms, though this has not been investigated. These factors are also likely to be much reduced in a domestic environment, especially with the use of rugs and supplementary feeding.

Within study herds there was notable variation in body condition, which varied between individuals on average by 1.8 BCS. In some herds individual BCS varied between 5 and 8/9, or between 4 and 7/9. Many of these highly variable herds were composed of the same breed types and were managed similarly. There is clearly more work to be done investigating body condition predictors in outdoor living animals, in particular a study which could quantify the amount of grass ingested and the nutritional quality of this forage in relation to body condition would be of value. In addition, we may not be able to generalise these results to horses and ponies stabled for much of the day or those undertaking increased levels of exercise; the risk factors for obesity in these individuals may be very different.

The estimates have important epidemiological strengths, not only was the prevalence measured in two separate seasons within the same population, but all measurements were taken by a single trained observer and obesity was defined and BCS categories grouped according to recent work exploring the validity of the BCS methodology (Dugdale et al., 2011).

The results highlight that seasonal variation in body condition is present within domestic equine populations and the extent of this seasonal variation appears to reduce as equines become obese. Future work is required to investigate the metabolic implications of a fluctuating versus constant body condition and to explore the associated health and welfare consequences.

## Supplemental Information

10.7717/peerj.299/supp-1 Supplemental Information 1 Copy of Winter Questionnaire

10.7717/peerj.299/supp-2 Supplemental Information 2 Copy of Summer Questionnaire

10.7717/peerj.299/supp-3 Supplemental Information 3 Paragraph regarding additional prevalence and risk factor estimates
