# Supplementary material for: Obesity prevalence and associated risk factors in outdoor living domestic horses and ponies
Source: PeerJ. 2014 Mar 20;2:e299. doi: 10.7717/peerj.299 (PMC3970797; doi:10.7717/peerj.299)
Supplement: Supplemental Information 1 [file peerj-02-299-s001.pdf]

Herd Number

Herd/Yard Questionnaire

1) How many horses/ponies in total are kept on this yard? ☐

2) How many horses/ponies are there in this herd[1]? ☐

3) What time of day are the horses turned out together?

Day ☐
Night ☐
Both ☐

4) For how many hours are all members of the herd out at pasture together currently? ☐

Please describe if necessary

____
____
____
____
____

5) Does this vary depending on the time of year?

Yes ☐
No ☐

[1] Herd refers to horses/ponies turned out together in the same field

If yes, by how much?

____

____

____

____

6) Does the herd as a whole get any additional feed when out at pasture at this time of year? E.g. Hay

Yes ☐

No ☐ If no go to question 8

If yes, please describe

____

____

____

____

7) How is this additional feed distributed within the paddock?

All in one large pile/feed manger ☐

Spread around the paddock ☐

Given specifically to each individual horse ☐

Don't know ☐

8) Do you ‘poo pick’ your horses pasture?

- Never ☐
- Occasionally ☐
- Most days ☐
- Everyday ☐

Individual Number	Herd Number

Individual Horse Questionnaire

1) What is your horse's/ponies' name? ____

2) What is your horse/pony's age (years/months)? [/] Please tick if this is an estimate

3) Which breed is your horse/pony? (if unknown state type) -

____

4) Which gender is your horse/pony?

Gelding ☐
Mare ☐
Stallion ☐

5) For how many years have you owned this particular horse/pony? ☐

6) Is your horse pregnant?

Yes ☐
No ☐
Don't know ☐

7) For how many years has this horse/pony been a member of this current grazing herd? ☐

8) How many hours of exercise, **per week**, on average, does your horse or pony get at this time of year? ☐

9) For **every hour** of exercise carried out, how many minutes, on average (best estimate), is spent carrying out the following activities?

**Activity**	**Time in Minutes (max 60)**
Walk	
Trot	
Canter	
Gallop	
Show Jumping	
Medium Dressage Movements	
Advanced Dressage Movements	
Cross Country Jumping	
Hunting	
Other	

Please state____________________________________

10) Do you ride your horse/pony competitively? If so in which events?

Yes ☐

No ☐

Which events?

____

____

____

____

11) For how many hours **<u>per day</u>**, on average, is this particular horse/pony out at pasture? ☐

12) Is your horse stabled (no dividing walls) with any other horses/ponies? If so, how many?

No ☐

Yes ☐ how many? ☐

13) Is this horse's/pony's grazing restricted in any way? E.g. by a grazing muzzle, restricted grazing etc

Yes ☐ please describe -

____

No ☐

14) How often does your horse/pony wear a rug when **out at pasture** at this time of year?

Everyday ☐

Most days ☐

Occasionally ☐

Never ☐

Which type of rug? Please circle (circle more than one if worn together).

Heavyweight   Medium weight   Lightweight   Stable Rug   Sunsheet

Rainsheet (waterproof)   New Zealand Rug   Fleece   Other under-rug

15) How often does your horse/pony wear a rug when **in the stable** at this time of year?

Everyday ☐

Most days ☐

Occasionally ☐

Never ☐

Which type of rug? Please circle (circle more than one if worn together).

Heavyweight   Medium weight   Lightweight   Stable Rug   Sunsheet

Rainsheet (waterproof) New Zealand Rug Fleece Other

under-rug

16) If any, what additional feed do you give this horse/pony each day? Tick all that apply

None ☐

Hay/Haylage/Silage ☐

Straw ☐

Sugar Beet ☐

Other root vegetables ☐

Concentrate/dry Feed ☐

Supplements ☐

Treats/titbits (specify type and frequency below) ☐

Please specify type and amounts in kg (if known) below

**Type**	**Amount (kg)**

17) Do you worm your horse? If so, how often?

No ☐

Yes ☐ how often?

____

18) Does your horse/pony have any history of injury or illness?

Yes ☐

No ☐

Not Sure ☐ (if no go to question 18)

19) If you answered yes above, tick all that apply

Respiratory Illness ☐

Laminitis ☐

Cushing's Disease ☐

Colic ☐

Musculoskeletal ☐

Other ☐

Please provide details (severity, frequency etc)

____

____

____

____

____

20) Does your horse/pony carry out any of the following behaviours when stabled? Tick those that apply

	Never	Occasionally	Most days	Everyday
Box Walking	☐	☐	☐	☐
Crib Biting/Wind sucking	☐	☐	☐	☐
Wood Chewing (separate from crib biting)	☐	☐	☐	☐
Weaving	☐	☐	☐	☐

21) Which bedding material do you use for your horse in the stable?

- Straw ☐
- Sawdust/Woodchips ☐
- Paper ☐
- Sand ☐
- Rubber Matting ☐
- Other ☐ please state ________________________________
