# Supplementary material for: Obesity prevalence and associated risk factors in outdoor living domestic horses and ponies
Source: PeerJ. 2014 Mar 20;2:e299. doi: 10.7717/peerj.299 (PMC3970797; doi:10.7717/peerj.299)
Supplement: Supplemental Information 2 [file peerj-02-299-s002.pdf]

| Individual Numbers | Herd Number |
| --- | --- |
| | |

## Summer Questionnaire

This questionnaire is mainly concerned with establishing how the management routine of your horse or pony has changed between winter and summer.

Name of horses/ponies in herd

________________________________________

Name of owner (will be treated as confidential)

________________________________________________

## Section 1 - Herd

*(as before please complete 'herd' section just once for each grazing group, but please complete a separate 'individual' section for each horse)*

**H1**) Has your horse moved into a completely new grazing herd since our last visit in February/March?

Yes ☐ Go to question H3

No ☐

**H2**) If no, have any horses left/joined the herd since the last visit?

No ☐

Yes ☐

If yes, please describe how many horses and state the months in which this occurred

______________________________________________________________________

______________________________________________________________________

______________________________________________________________________

______________________________________________________________________

_________________________________________

**H3**) How many horses are now in the herd? ☐

**H4**) Does the herd as a whole get any additional feed when out at pasture at this time of year?

Yes ☐ Please specify ________________________________________________

No ☐ (if no, go to question H6)

**H5**) If yes, how is this food distributed?

Spread Around ☐

Fed in one large pile ☐

Given to each individual ☐

**H6**) How many times have the herd changed turnout pasture (moved paddock) since the last visit (February/March)? ☐

**H7**) Are parts of the pasture fenced off at any point in time? I.e. do you implement strip grazing or something equivalent?

Yes ☐

No ☐

| Individual Number | Herd Number |
| --- | --- |
| | |

**Section 2 – Individual**

Name of horse/pony ____________________________________________

Section A - Turnout Routine

**A1**) Has this particular horse experienced any change in herd since the last visit? (horses left, arrived or a complete change)

No ☐

Yes ☐ please describe

__________________________________________________________________________

__________________________________________________________________________

__________________________________________________________________________

_________________________________

__________________________________________________________________________

___________

**A2**) For how many hours per day is your horse/pony out at pasture at this time of year?

☐

**A3**) Do you actively monitor the weight of your horse/pony?

No ☐
Yes, all year ☐
Yes, summer only ☐
Yes, winter only ☐

If yes, please state how

______________________________________________________________________
______________________________________________________________________
______________________________________________________________________
______________________________________________________________________
__________________________________________

**A4**) Do you make an effort to actively control the grass intake of your horse/pony during the summer months?

Yes ☐
No ☐ (if no go to section B)

**A5**) If yes, which if the below best describes how this is achieved (tick all that apply)?

Moving horses onto less/more lush pasture ☐
Grazing Muzzle ☐
Restricted number of grazing hours ☐

Removal from pasture altogether (e.g. kept in stable) ☐

Other (please state) ________________________ ☐

**A6**) Please describe this summer routine in detail (e.g. for how many hours is the horse/pony off grass, how many times or in which situation horses move pasture etc)

______________________________________________________________________

______________________________________________________________________

______________________________________________________________________

______________________________________________________________________

______________________________________________________________________

______________________________________________________________________

______________________________________________________________________

______________________________________________________________________

___

**A7**) During which month(s) did you begin each aspect of this summer management routine (please describe)?

______________________________________________________________________

______________________________________________________________________

______________________________________________________________________

______________________________________________________________________

______________________________________________________________________

______________________________________________________

.Section B - Exercise

**B1**) How many hours of exercise, **per week**, on average, does your horse or pony get at this time of year?

**B2**) For **every hour** of exercise carried out, how many minutes, on average (best estimate), is spent carrying out the following activities?

| **Activity** | **Time in Minutes (max 60)** |
|---|---|
| Walk | |
| Trot | |
| Canter | |
| Gallop | |
| Show Jumping | |
| Medium Dressage Movements | |
| Advanced Dressage Movements | |
| Cross Country Jumping | |
| Hunting | |

| | |
|---|---|
| Other | |

Please state_________________________________

## Section C - Feed

**C1**) Do you change what you feed your horse/pony between the summer and winter months? Please tick most applicable

No change (go to section D) ☐
Change in the quantity fed but not the diet ☐
Change parts of the diet between seasons ☐
Complete change in diet between seasons ☐

**C2**) If any, what additional feed do you give this horse/pony each day at this time of year? Tick all that apply

None ☐
Hay/Haylage/Silage ☐
Straw ☐
Sugar Beet ☐
Other root vegetables ☐
Concentrate/dry Feed ☐
Supplements ☐
Treats/titbits (specify type and frequency below) ☐

Please specify type and amounts of dry feed in kg (if known) below

| **Type** | **Amount (kg)** |
|---|---|
| | |
| | |

| | |
|---|---|
| | |
| | |
| | |
| | |
| | |

## Section D - Health

**D1**) Is your horse or pony pregnant?

No ☐

Yes ☐

Not Sure ☐

**D2**) Have there been any new injuries or illnesses since the winter questionnaire was completed in February/March?

No ☐

Yes ☐ please describe

______________________________________________________

**D3**) To your knowledge, has your horse/pony ever had any dental problems?

No ☐

Yes ☐ please describe

______________________________________________________

**D4**) Has your horse or pony ever had any dental treatment e.g. rasping?

Yes ☐

No ☐ If no, go to question D6

**D5**) If yes, when was this? (rough date) ____________________________________________

**D6**) If no, have your horses teeth ever been examined by a professional?

Yes ☐

No ☐

Section E - Rugs

**E1**) How often does your horse/pony wear a rug when **out at pasture** at this time of year?

- Everyday ☐
- Most days ☐
- Occasionally ☐
- Never ☐

Which type of rug? Please circle (circle more than one if worn together).

Heavyweight    Medium weight    Lightweight    Sunsheet/flysheet

Rainsheet (waterproof)    New Zealand Rug    Fleece

Other under-rug    Stable Rug

**E2**) How often does your horse/pony wear a rug when **in the stable** at this time of year?

- Everyday ☐
- Most days ☐
- Occasionally ☐
- Never ☐

Which type of rug? Please circle (circle more than one if worn together).

Heavyweight Medium weight Lightweight Sunsheet/flysheet

Rainsheet (waterproof) New Zealand Rug Fleece

Other under-rug Stable Rug
