# Supplementary material for: Obesity prevalence and associated risk factors in outdoor living domestic horses and ponies
Source: PeerJ. 2014 Mar 20;2:e299. doi: 10.7717/peerj.299 (PMC3970797; doi:10.7717/peerj.299)
Supplement: Supplemental Information 3 [file peerj-02-299-s003.pdf]

Supplementary Information 3: Paragraph regarding additional prevalence and risk factor estimates

Of additional interest to readers is that we calculated the prevalence of obesity on the initial 127 horses recruited at the end of winter and the prevalence estimate was 27.56% (95% CI 19.79% - 35.32%). At the end of the summer months we also measured the body condition of new horses and ponies that had entered our original herds (n=43, giving a total summer n=139). The prevalence of obesity at the end of the summer in these animals was 34.53% (95% CI 28.87% - 40.19%). These estimates present a larger sample size and more precise prevalence estimates (reassuringly very similar to those with the 96 animals measured during both seasons). These are not appropriate to present within the main paper due to our study design and associated risk factor analysis. When risk factors were assessed separately on these winter and summer populations they were identical to those within the paper, except that they did not include ‘new injury between winter and summer’.
